# Spinopelvic Dissociation Case Report and Literature Review

**DOI:** 10.7759/cureus.39750

**Published:** 2023-05-30

**Authors:** Nouf A Altwaijri, Mohammed Abdulaziz, Rafiq Bhat, Ahmad Ellafi, Khaled A Alhabdan

**Affiliations:** 1 Orthopedic Surgery, King Saud Medical City, Riyadh, SAU; 2 College of Medicine, King Saud Bin Abdulaziz University for Health Sciences, Riyadh, SAU

**Keywords:** incomplete spinal cord injury, cauda equina syndrome, lumbar burst fractures, listhesis, spinopelvic dissociation

## Abstract

A 32-year-old male was brought to our emergency department following trauma due to fall of heavy object (tree) on his back. After Advanced Trauma Life Support (ATLS) protocol implementation, the patient was noted to have a complete perianal tear and loss of power in L3-S1 measuring 1/5 complete loss of sensation below the level of L2. Imaging showed spinopelvic dissociation with cauda equina syndrome. Spinopelvic fixation and fusion with rigid fixation done. The patient regained normal function following extensive physiotherapy. This paper concludes that good and prompt surgical intervention facilitated neurological recovery following decompression.

## Introduction

Spinopelvic dissociation is defined as having a transverse sacral fracture alongside a sagittal fracture of the sacrum occurring bilaterally, causing dissociation of the spine and upper sacrum from the pelvis [1-4]. Gravity and the hip flexors cause flexion of the distal segment relative to the proximal on the sagittal plane, resulting in kyphosis deformity in the fracture site [5,6]. In spinopelvic dissociation, sacral fractures can be H-, U-, or Y-shaped [7,8].

Sacral fractures rarely occur on their own, as they usually occur alongside other fractures of the pelvic ring. Spinopelvic dissociation is related mostly to high-energy trauma in adults [9,10]. Given the nature of the trauma that causes these types of fractures, they are associated with various neurological injuries such as cauda equina syndrome, plexus, or radicular injuries [11,12]. In addition to local soft-tissue damage, visceral organ damage, or injury, other fractures and hemorrhages can occur [1,2,4,13]. Although these fractures can be missed due to the severity of other associated injuries, such as head or thoracoabdominal trauma, it is of upmost importance to establish the presence of spinopelvic dissociation accurately and efficiently for proper management and to avoid the consequences of progressive deformity and chronic pain [11,12,14,15].

There are two classification systems commonly used for spinopelvic dissociation classification, Denis classification and Roy-Camille classification, which were adjusted by Strange-Vognsen and Lebech [1,3,10,16]. The Denis classification system comprises three zones: fractures occurring lateral to the sacral neuroforamina (zone I), fractures involving the neural foramina without the spinal canal (zone II), and fractures extending to the neural canal (zone III) [16,17].

The Roy-Camille system classifies sacral fractures into three types: flexion fracture with anterior bending of upper fragments of the sacrum (type I); flexion fracture with posterior upper fragment displacement where it settles itself on the lower fragment of the fracture and becomes more or less horizontal (type II); and extension fracture with an upper fragment anterior displacement that slips downward in front of the lower fragment and becomes more or less vertical (type III) [10]. Roy-Camille describes spinopelvic dissociation as the “suicidal jumper fracture,” given that these fractures are commonly caused by jumping from a height in a suicide attempt [1,10]. These classifications aid in the prediction of neurological deficit and functional outcome [1,3,10,16].

## Case presentation

**Figure 1:**
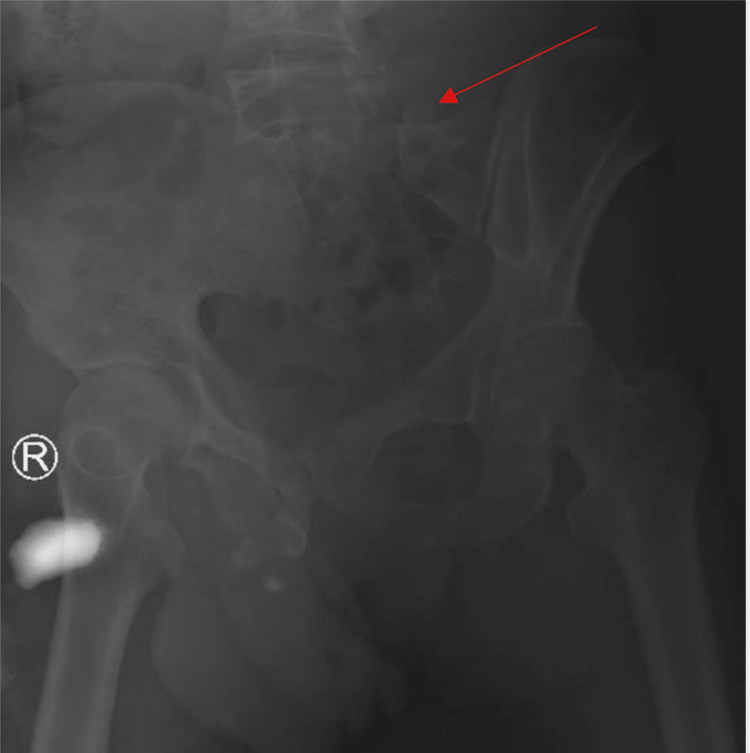
Anteroposterior (AP) X-ray of the pelvis. The arrow shows transforaminal fracture of the left sacrum (Denis type II) with proximal migration and displacement.

A 32-year-old man was brought to our emergency department in a fully conscious state with a Glasgow Coma Scale (GCS) score of 15/15 following trauma due to a heavy object (tree) falling on his back while working on a farm. The patient was assessed and managed as per the Advanced Trauma Life Support (ATLS) protocol. Once stabilized, a detailed examination was done, which revealed skin contusion over the lower back and complete perianal tear with mild abdominal distention and tenderness. A neurological examination revealed intact motor and sensory examination in the upper limbs. However, in the lower limbs, power was 1/5 from L3-S1, and there was a complete loss of sensation below the L2 level. Perianal sensations and anal tone could not be examined due to the complete perineal tear and because the patient was on a Foley catheter. Examination was done according to the American Spinal Injury Association Impairment (ASIA) scale. An X-ray of the pelvis is shown in Figure 1.

**Figure 2:**
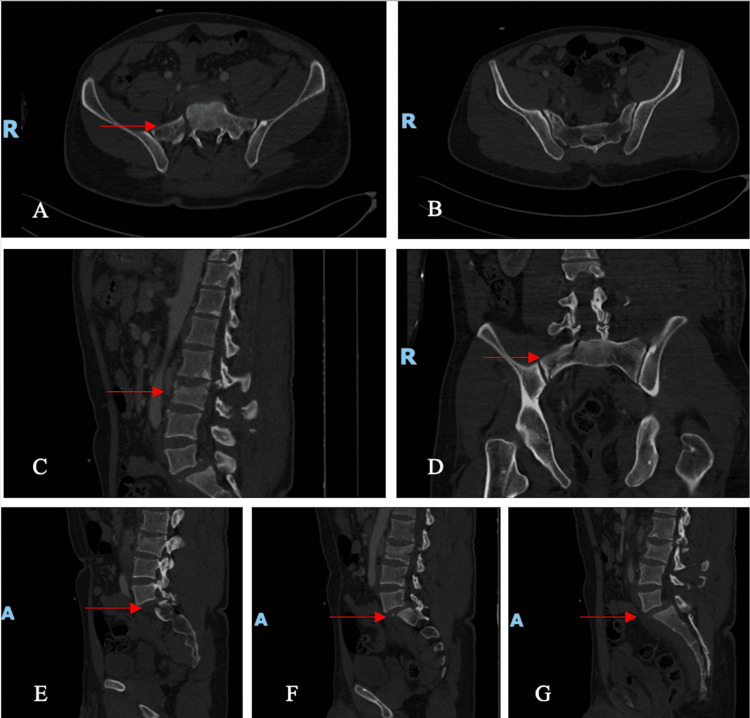
CT scan of the pelvis and spine showing pelvic dissociation, sacral ala fracture, L3 fracture and facet dislocation and listhesis of L5-S1 A, B: Axial cuts. The arrows show sacral ala fracture; C: Sagittal cut. The arrows show L3 fracture; D: Coronal cut. The arrow shows sacral ala fracture; E, F, G: Sagittal cuts. The arrows show L5-S1 facet dislocation and listhesis

A CT scan of the spine and pelvis indicated a burst fracture of L3 with retropulsion and L5-S1 dislocation with bilateral locked L5-S1 facets and spondylolisthesis grade 3 [18]. This is seen in Figure 2.

 

**Figure 3:**
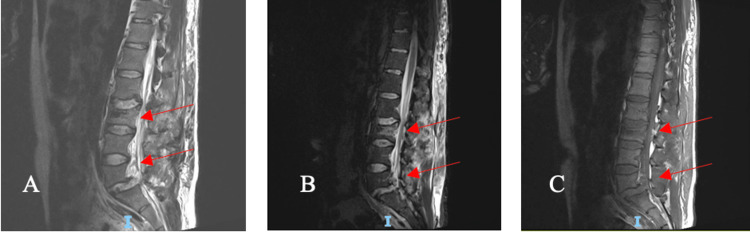
Findings of an L3 fracture with posterior ligamentous complex injury with intrathecal bony fragment with L5-S1 listhesis pointed to by the arrows on different cuts (sagittal T2, STIR and T1 sagittal cuts)

Moreover, there was a sacral ala fracture in addition to abdominal, pre-sacral, and back soft-tissue hematomas. An MRI examination of the patient showed L3 fracture with posterior ligamentous complex injury with intrathecal bony fragment with L5-S1 listhesis (Figure 3).

**Figure 4:**
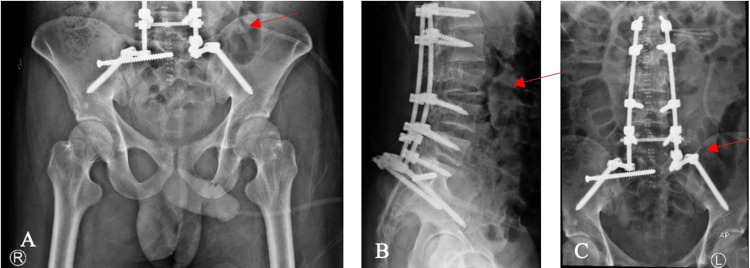
Post fixation X-rays A: Anteroposterior (AP) view of the pelvis; B: Lateral; C: AP view of fixation. The arrows show the fixation.

After initial stabilization and preparation for operative fixation, the patient was taken to the operating room (OR) for perineal repair with a diversion colostomy and a stoma to allow for the complete healing of the perineal tear. Afterwards, the patient was then planned for and taken to the OR for fixation. The patient was put in the prone position, and, after prepping and draping in a sterile manner, approached with a midline posterior incision for posterior spino-pelvic fixation and fusion with a triangular rigid fixation from L1 to ileum with decompression at L3 and L5 levels using percutaneous sacroiliac fixation [19,20]. This is seen in Figure 4.

**Figure 5:**
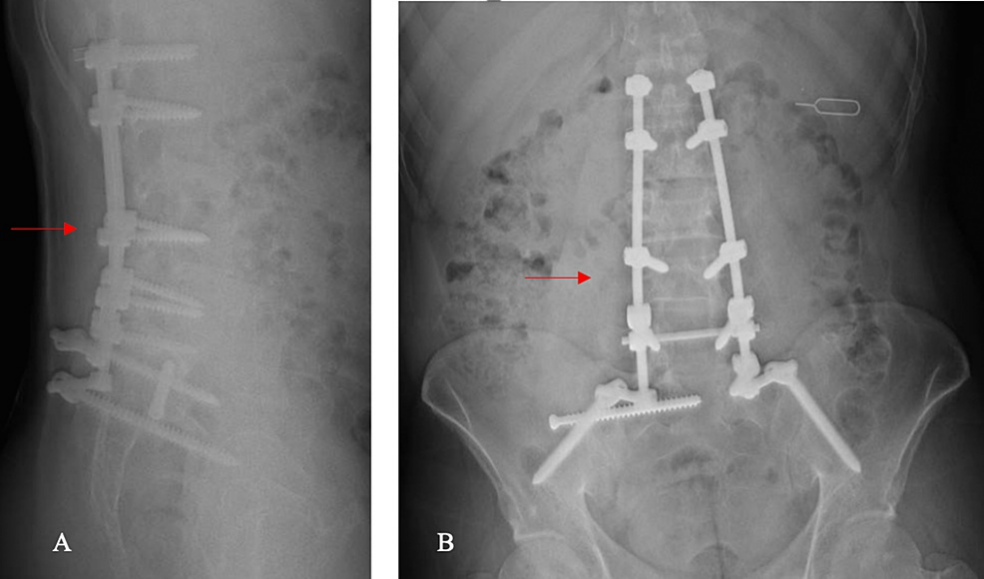
X-rays at one-year follow-up A: Lateral; B: Anteroposterior (AP). The arrows show stable fixation at follow-up.

Dura was found to be torn at the L3 level and repaired using 6.0 Nylon sutures. Postoperatively, the patient was doing well and was put on a comprehensive spinal-cord-injury rehabilitation program. Postoperative examination revealed slow improvement in his neurological status with rehabilitation. The patient was again examined with a radiological examination to see the status of fixation at six and 12 weeks, which revealed a satisfactory outcome. After six months postoperatively, the patient was noted to regain full neurological function and complete recovery of his motor power in L3-S1 (5/5) and sensation (2/2), and he was admitted again for stoma reversal, where per rectal examination was intact. At one-year follow-up, the patient was doing well and had completed rehabilitation. There was no implant failure, and the wound healed satisfactorily. He regained full neurological function and was fully able to go back to his daily activities. X-rays at the one-year follow-up are shown in Figure 5.

Informed consent was obtained from the patient prior to publication of this study.

## Discussion

We present a case of spinopelvic dissociation where there was an L5-S1 dislocation, spondylisthesis grade 3, sacral ala fracture, and severe cauda equina that was managed operatively with rigid triangular fixation using iliolumbar reduction and decompression, which yielded excellent results. For such fractures, no specific treatment paradigm is available, and management is case by case. Lehman et al. proposed an algorithm for non-operative management where non-operative treatment can be considered if the patient is likely to be bed-bound or non-weight bearing for three months [21,22]. However, if the degree of kyphosis in these fractures is greater than 20 degrees, it is likely to progress and cause more instability, and, therefore, surgery must be considered [21,22].

Sullivan et al. concluded that the current standard of care for such fractures is triangular osteosynthesis and iliosacral screw osteosynthesis [21]. Schildhauer et al. concluded that lumbopelvic fixation provides good stability and permits consistent union of the fracture without alignment loss [12]. Chen et al. managed a U-shaped sacral fracture with cauda equina through posterior sacral laminectomy with satisfactory results [23]. Tahir et al. studied multiple cases of displaced spinopelvic dissociation with cauda equina syndrome and concluded that surgical decompression and lumbopelvic segmental fixation enhances neurological recovery and provides good structural stability with a good clinical outcome [24]. Schildhauer et al. noted that lumbopelvic fixation provides dependable stability for the fracture, allowing for fracture union with maintained alignment, whereas the neurological outcome depends on the injury and sacral root disruption [12].

Nerve root injury is one of the factors that have a major influence on short- and long-term functional outcome [1,7,25]. Petryla et al. studied the functional outcome and quality of life of patients following surgical management of such fractures and noted that most patients with neurological deficits had improved neurological status following surgery. Moreover, their study noted a decrease in physical activity at one year following surgery, with no changes observed in mental status [1]. Lindahl et al. studied the factors that affected the outcome and noted that complete translational displacement of the sacral transverse fracture was associated with a poor outcome, whereas partial translation was associated with a good outcome [7]. Their results also indicated that the final outcome was related to the degree of initial transverse sacral segment translational displacement, where permanent cauda equina symptoms and lumbosacral plexus injury were associated with complete transverse segment displacement [7]. A systemic review by Patel et al. concluded that spinopelvic fixation in patients with unstable vertical pelvic fractures and sacral fractures with spinopelvic dissociation is an effective method with good radiological and functional outcomes [26]. However, sufficient precautions are required to avoid infections and wound complications [26]. 

## Conclusions

Spinopelvic dissociation injuries following trauma are extremely rare injuries that require expeditious management. Given the nature of the trauma that leads to these types of fractures, they are associated with various neurological injuries such as cauda equina syndrome, plexus, or radicular injuries, which is why it is vital to ensure timely intervention. This case report highlights how good and prompt surgical intervention facilitates neurological recovery following decompression with the aid of an extensive rehabilitation program following surgery and continuous clinical follow-up. 
